# Editorial: Neuromodulatory Control of Brainstem Function in Health and Disease

**DOI:** 10.3389/fnins.2020.00086

**Published:** 2020-02-11

**Authors:** Brian R. Noga, Ioan Opris, Mikhail A. Lebedev, Gordon S. Mitchell

**Affiliations:** ^1^Miller School of Medicine, University of Miami, Miami, FL, United States; ^2^Department of Biomedical Engineering, University of Miami, Coral Gables, FL, United States; ^3^Department of Neurobiology, Duke University, Durham, NC, United States; ^4^Center for Bioelectric Interfaces of the Institute for Cognitive Neuroscience, National Research University Higher School of Economics, Moscow, Russia; ^5^Department of Information and Internet Technologies of Digital Health Institute, I.M. Sechenov First Moscow State Medical University, Moscow, Russia; ^6^Center for Respiratory Research and Rehabilitation, Department of Physical Therapy, University of Florida, Gainesville, FL, United States

**Keywords:** brainstem, neuromodulation, locomotion, neurotransmitters and motor control, autonomic function, spinal cord injury, movement-related disorders, pain

The brainstem plays a crucial role in the control of locomotion, posture, balance, arousal (alertness, awareness, and consciousness), sensory information processing, respiration, autonomic functions (including control of blood pressure, heart rate, bowel, and bladder), and is responsible for the regulation of multiple reflexes including coughing, swallowing, and vomiting. It is controlled by the executive centers in the brain originating from cortical areas and subcortical regions, including the basal ganglia nuclei and the diencephalon, as well as, feedback loops from the cerebellum and spinal cord. A modulatory control of brainstem output can be accomplished by affecting single neurons and consequently, the operation of neuronal microcircuits and behavior. This is accomplished by altering cellular excitability, synaptic transmission (release probability, postsynaptic receptor responsiveness, thus altering synaptic strength, and efficacy) and network properties. Such dynamic control provides flexibility to the brain systems to adapt their outputs in synchrony to the functional requirements/demands of the individual to achieve the desired behavioral goal in a changing environment. Classical ionotropic transmitters: glutamate/acetylcholine, glycine/GABA (gamma-amino butyric acid) are responsible for the primary excitation and inhibition of the “anatomical network.” In addition, transmitters such as the monoamines (serotonin, dopamine, or noradrenaline), acetylcholine, glutamate and GABA can alter electrical and synaptic properties of neurons by acting on metabotropic (G protein-coupled) receptors which affect signal transduction pathways.

This special topic on *Neuromodulatory Control of Brainstem Function in Health and Disease*, highlights recent advances in our understanding of the intrinsic and extrinsic neuromodulatory systems affecting brainstem function and means to control them. Two hundred twenty-five authors contributed 35 articles to this Research Topic. The contributions are summarized below in 9 thematic categories: (i) motor control, (ii) neurotransmitters and motor control, (iii) cardiovascular, respiratory and other autonomic functions, (iv) movement-related disorders, (v) pain, (vi) other disorders, (vii) non-invasive stimulation approaches for modulation of brainstem function, (viii) brain computer/machine interfaces (BCI), and (ix) non-stimulation approaches.

## Motor Control

A group of experimental studies performed in a variety of animal models provide new insights into specific brainstem circuits involved in locomotion, posture, and voluntary (reach-to-grasp) movements.

Opris et al. examined the distribution of locomotor-activated neurons along the brainstem, combining c-Fos immunohistochemistry and cellular phenotyping. Locomotion was induced by electrical stimulation of the mesencephalic locomotor region (MLR). Of the putative anatomical correlates of the physiologically defined MLR [the cuneiform nucleus (CnF) and the pedunculopontine nucleus (PPN)], only neurons within the CnF showed significant Fos labeling, supporting the idea that the CnF is the anatomical correlate of the MLR. Local field potentials (LFPs) are thought to coherently bind cooperating neuronal ensembles during the production of different behaviors and may be useful as biomarkers to target brain regions for deep brain stimulation (DBS). Noga et al. recorded LFPs within previously identified MLR sites during voluntary locomotion. In low threshold MLR stimulation sites, onset and speed of locomotion was best correlated to the appearance and power of theta rhythms. The results demonstrate that theta band activity may be a suitable biomarker to identify functional MLR sites. The MLR is but one node or integration center involved in the descending control of spinal locomotor circuits and the operational node used at any one time is context dependent. By integrating work from the locomotion and animal behavioral domains, Kim et al. examined the neural circuits for context-specific control of locomotion, as well as approach and avoidance behaviors affecting onset and offset of locomotion. Special emphasis was given to the descending modulatory control of spinal locomotion centers from the MLR and the diencephalon. Using optogenetic stimulation, Koblinger et al. demonstrate that cells within the dopaminergic A11 region of the posterior diencephalon can enhance motor activity which may lead to locomotion.

Efficient locomotion depends upon proper control of both hindlimb and trunk muscles. The control of trunk musculature by the brainstem was examined by Jean-Xavier and Perreault using calcium imaging to discern activation of axial and hindlimb motoneurons during pharmacologically induced locomotion in the isolated neonatal mouse brainstem-spinal cord preparation. Removal of the brainstem resulted in an increase in locomotor rhythm frequency and a concurrent reduction in motoneuron burst durations, suggesting that the brainstem plays a central role in the control of trunk activity during walking.

The reticular formation is important for the integration of descending inputs controlling movement. The review by Brownstone and Chopek examines the areas of the pontomedullary reticular formation that control posture, walking and sleep and their role in the activation and inhibition of movement.

Precision reach-to-grasp movements require coordination of proximal and distal muscles controlling shoulder, arm and hand. The role of the cerebellum in reach-to-grasp movements was investigated by Geed et al. The results indicate that there is a correlation of activity between similar functional ensembles within the cerebellar nucleus interpositus and its target, the magnocellular red nucleus, which act synergistically to control complex coordinated movements.

## Neurotransmitters And Motor Control

Another group of papers highlight the role of the neurotransmitters dopamine, noradrenaline, serotonin, and histamine in modulating the electrical and synaptic properties of brainstem neurons during motor control.

Reticulospinal neurons are known to convey the descending command for the initiation of locomotion with stimulation of the MLR. Opris et al. show that stimulation of the MLR also activates serotonergic and catecholaminergic neurons of the pons/medulla, in addition to reticulospinal neurons. The study provides anatomical and functional evidence for spinal monoamine release during evoked locomotion. Koblinger et al. provide evidence for the existence of a new catecholaminergic neuron subtype within the A11 region, that contributes to the control of motor activity. Their data suggests that this catecholaminergic motor circuit forms part of the diencephalic locomotor region. Dopaminergic neurons modulate locomotion via their projections to the basal ganglia which ultimately affect the brainstem locomotor networks. Ryczko and Dubuc review new findings that indicate dopamine neurons project to the MLR in many vertebrate species. They summarize studies demonstrating that dopamine is released in the MLR and modulates neuronal activity there by acting on D1 receptors.

There is increasing evidence for the involvement of histamine in the modulation of motor responses. Li B. et al. show that histamine, acting on the H2 receptor, increases the excitability, and sensitivity of lateral vestibular neurons and contributes to improved central vestibular-mediated motor behaviors.

## Cardiovascular, Respiratory, And Other Autonomic Functions

Several studies discuss new findings concerning the neuromodulatory control of respiratory, vestibular, cardiovascular and micturition systems.

Opris et al. discuss how these systems are coupled during MLR-evoked locomotion. Airway resistance is modulated by vagal preganglionic (AVP) neurons, which are regulated by thyrotropin-releasing hormone (TRH). Hou et al. show that TRH regulates inspiratory-activated AVP neuronal activity by: (i) modulating the response to excitatory and inhibitory inputs; (ii) activation of an excitatory postsynaptic slow inward current; and (iii) production of a gap junction-mediated oscillatory pattern of activity. Central chemoreception of changes in hydrogen ion concentration within the brain help to regulate respiration and acid-base homeostasis. Wang X et al. show that local pH changes within the ventrolateral medulla modulate breathing not only by acid-sensing ion channels (voltage-independent proton-gated cation channels) but by a novel mechanism utilizing TWIK-related acid-sensitive potassium channels.

When swallowing occurs at an inappropriate time during inspiration, it increases the possibility of aspiration which can cause dysphagia. Yagi et al. investigated respiratory and swallowing activity in patients with dysphagia vs. normal subjects. Their results show a direct correlation between breathing–swallowing discoordination and the severity of dysphagia.

Sympathetic premotor neurons of the rostral ventrolateral medulla (RVLM) play an important role in the generation of vasomotor sympathetic tone. Dempsey et al. mapped the inputs to the spinally projecting RVLM neurons and found that the majority of inputs originate within the brainstem. These are likely to control respiratory-sympathetic coupling. Angiotensin II is a powerful vasoconstrictor that interacts with glutamate and GABA within the RVLM to modulate blood pressure. Légat et al. found that selective stimulation of angiotensin II type 2 receptors within the RVLM in normotensive rats increases local GABA levels and decreases blood pressure. Jiang et al. found that angiotensin II also mediates the pressor effect through a specific intracellular signaling mechanism in the glutamatergic neurons of the RVLM in rats with stress-induced hypertension.

The brainstem has a crucial role in micturition. Segmental afferent input from the skin of the perineum is known to inhibit bladder contractions due to its effects on signal transmissions between the brain and spinal cord. Hotta et al. examined the hypothesis that an overactive bladder in old age may be due to a malfunction in this inhibitory mechanism. Their results show that while the inhibitory mechanism is present in aged rats, there is evidence for reduced responses in Aδ- and C-low-threshold mechanoreceptors that can explain the weak inhibition observed in older rats.

## Movement-Related Disorders

A group of studies focused on movement-related disorders provide valuable insight into gait impairments, muscle weakness, postural control and motor excitability in disorders resulting from focal ischemic stroke, hemiparetic stroke, post-stroke hemiplegic gait, Parkinson disease (PD), or restless legs syndrome.

Gait impairments are common after stroke due to the development of spasticity and paresis. Li S. et al. provide a new perspective and insight into hemiplegic gait. Damage to the motor cortex and the corticospinal tracts leads to muscle weakness. Descending brainstem and intraspinal circuits controlling posture and locomotion are disinhibited causing hyperexcitability and spasticity. These changes lead to a reorganization of the spatiotemporal patterns of activation of limb and trunk muscles during gait. They propose that post-stroke hemiplegic gait is the result of the mechanical consequences of this reorganization. This new perspective has important clinical implications for the management of hemiplegic gait. Changes in the descending neural drive (like those in focal ischemic stroke) have the potential to alter the spinal motor excitability, that may increase in the chronic hemiparetic stroke state, inducing the exaggerated stretch-sensitive reflexes. To infer the sources of spinal motor excitability in individuals with chronic hemiparetic stroke, McPherson et al. examined tonic vibration reflexes during voluntary muscle contractions. Their findings indicate that the increased excitability of motor neural ensembles innervating the paretic limb may come from neuromodulatory and ionotropic mechanisms.

In Parkinson Disease, DBS of the PPN or the subthalamic nucleus significantly improves motor symptoms and postural instability. As a treatment alternative to DBS, Cakmak et al. used intrinsic auricular muscle zone stimulation to modulate the activity of these motor centers. Clinically significant improvements in motor function were observed following stimulation indicating that such approach may be useful as a minimally invasive approach to treat PD. Ryczko and Dubuc provide a review of the involvement of dopaminergic neurons in the activation of neurons within the MLR and ultimately reticulospinal neurons, for the control of spinal locomotion. The potential involvement of dopaminergic neurons in the pathology of PD is discussed. On the other hand, the long-term use of levodopa (l-dopa) for the treatment of PD results in multiple adverse effects including drug-induced dyskinesias. Adenosine may alleviate L-dopa induced dyskinesias (LID) but the mechanism of this effect is unknown. Wang W-W. et al. conducted a metanalysis of the efficacy of adenosine A2A receptor antagonists in reducing LID. They found that although these drugs have efficacy in animal models of LID, more studies are warranted to establish this approach. In another study of PD (Miroshnichenko et al.) found that motor symptoms of PD (elevated muscle tone) were relieved with dry immersion of subjects (wrapped in a waterproof film) in water to deprive muscles of the sensory stimuli that activate reflexes. The results indicate its potential use as a rehabilitation strategy for PD patients.

Restless legs syndrome (RLS) is a sensorimotor disorder characterized by an increased urge to move the legs when they are at rest. RLS may be successfully treated using agonists that target the inhibitory dopamine D3 receptor subtype. Although there is little evidence in RSL patients of D3 receptor dysfunction, there is evidence that a mutation in the MEIS1 gene (which has been linked to an altered phenotype of dopaminergic neurons) increases the risk for developing RLS. Meneely et al. assessed the effects of dopaminergic treatment and spinal cord dopamine receptor expression in two different dopaminergic receptor knockout models of RLS. Their results suggest that the two models are complimentary and are best used to explore different aspects of RLS related sensory and motor dysfunction.

## Pain

A variety of neuronal systems within the brainstem are involved in the processing and modulation of pain signaling. These include the periaqueductal gray (PAG) and the monoaminergic nuclei, which comprise the descending pain inhibitory system. These nuclei are interconnected with higher brain centers involved in pain or nociceptive processing. A number of papers evaluated the effectiveness of different stimulation strategies aimed at reducing pain/nociception. Their results provide further evidence for the effectiveness of DBS, transcranial direct current stimulation (tDCS), and electroacupuncture (EA) for the treatment of chronic neuropathic pain.

Chronic neuropathic pain (CNP) is a major problem following SCI and few therapeutic approaches (from pharmacological to non-pharmacological) bring relief to CNP patients. Jermakowicz et al. reported the case of a patient with severe CNP (from the incomplete paraplegia), treated with bilateral DBS of the midbrain periaqueductal gray (PAG). Their results show that DBS of the PAG had a major effect on the severity of CNP and reversed the neurological abnormalities associated with pain. The findings further suggest that activation of endogenous pain inhibitory systems linked to the PAG, may remove CNP in many patients with SCI.

In a different study, Wen et al. evaluated the prolonged analgesic effects of tDCS of primary motor cortex (M1) on chronic neuropathic pain in rats. The reported anti-nociceptive effects of tDCS had a longer analgesic effect and depend on the intensity and time of stimulation. These findings are important to the clinical application of tDCS.

To relieve visceral pain, Yu et al. applied an efficient treatment—electroacupuncture. To document the effect, they recorded neuronal firing in the medullary subnucleus reticularis dorsalis of anesthetized rats. Their findings revealed that EA induced an inhibiting effect on visceral nociceptive signals, likely due to the somato-visceral interaction within these neurons.

## Other Disorders

Several brainstem reticular nuclei are involved in the control of sleep/wakefulness (consciousness) and in the control of nausea. Two papers provide insight into different strategies for the neuromodulation of disorders of consciousness and nausea.

Spinal cord stimulation may be used to treat patients with disorders of consciousness. Examining the effects of spinal stimulation on the connectivity/network properties of patients with minimally conscious state, Bai et al. confirmed that this strategy can affect gamma cortical activity, producing instant global effects (large scale connectivity and network alteration), as well as long-lasting local effects. The data indicates that the stimulation effects on consciousness may be the result of an altered frontal cortical-thalamo-cortical network function.

Median nerve stimulation (MNS) is known to alleviate the symptoms of nausea and vomiting. In a clinical trial, Maharjan et al. investigated the effect of MNS on human olfactory function, a major sensory modality for inducing vomiting and nausea. They show that only high frequency MNS can suppress odor perception. This method may be useful to treat nausea and malnutrition accompanying different health conditions.

## Non-Invasive Stimulation Approaches For Modulation Of Brainstem Function

Two experimental studies explore the utility of non-invasive stimulation approaches to modulate brainstem function: transcranial magnetic stimulation (TMS), based on the electric current induced by a change in magnetic field and focused ultrasound stimulation (FUS) using low intensity, low frequency ultrasound.

Chen et al. used TMS to evaluate the excitability of the laryngeal motor cortex and its responses to neuromodulation by measuring motor evoked potentials and the cortical silent period (cSP). Their results support the feasibility of using TMS for measuring laryngeal muscles responses during vocalization and thus provide a new tool for understanding the neural control of voice production.

Neuronal oscillations coordinate neural ensembles by coupling the firing of multiple neurons through phase-amplitude coupling (PAC). PAC is closely linked with cognitive brain functions and may be used to measure cortical excitability as well as network interactions. Yuan et al. examined the relationship between the intensity of ultrasounds and the PAC index in the rat brain. They demonstrated that FUS can be used to modulate neural firing with tremendous spatial precision leaving open the possibility that this method may be useful for improving cognitive abilities.

## Brain Computer/Machine Interfaces (BCI)

A brain computer interface (BCI) is a neural control system that provides a communication pathway between a computer, the brain and an external device/actuator. An interesting BCI is based on the concept of mirror neurons. A “mirror neuron” represents a special type of neuron that “mirrors” the behavior of an observer, assuming the observer was itself acting. The components of the human mirror neuron system activate subcortical systems and “sensorimotor” cortices (occipital or parietal) when a subject performs or observes an action. Engaging the mirror system in such a fashion can be used as a therapeutic strategy for rehabilitation after brain injury or disease. An “action observation” produces “event-related desynchronization” (ERD) suppressions in the human brain by activating regions of the “mirror neuron system.” Luo et al. examined the mirror system's response in different cortical regions to the speed of action observed by adjusting the movement speed of a robotic arm. Results indicate the possibility to construct BCIs based on patterns of action observation.

Li H. et al. built an EEG brain functional network with dynamic connections and used its special characteristics to derive synchronization features. These were used to interpret processing during a memory paradigm and examine differences between healthy controls and mental patients. The results provide new insights into the pathology of brain disorders.

## Non-Stimulation Approaches

Finally, a group of studies explore the utility of non-stimulation approaches to modulating brainstem function, examining the role of astrocytes in the locus ceruleus in pain resulting from emotional dysfunction, the antidepressant-like effect of algae on dopaminergic function and the regulation of gene expression by long non-coding RNAs in the pathophysiology of central nervous system diseases.

Emotional dysfunction joined by early life stress has been shown to increase the perception of pain. Nakamoto et al. examined the role of astrocytes in emotional dysfunction in mice under maternal separation and social isolation as a source of life stress. Their results show that astrocyte activation in the locus coeruleus is involved in the increase of neuropathic pain caused by maternal separation.

Algae have been shown to have a variety of beneficial effects on health. Sasaki et al. assessed its antidepressant effect using a forced-swimming test rodent model of depression. They found that an extract of the colonial green alga *Botryococcus braunii* enhanced the expression of genes involved in neurogenesis, energy metabolism and dopamine synthesis. They suggest that the antidepressant-like effect of this alga is due to enhanced dopaminergic function.

The review by Wei et al. describes the varied roles for long non-coding RNAs, and how they may be involved in the development of the etiology and pathophysiology of central nervous system diseases.

In conclusion, the Research Topic on *Neuromodulatory Control of Brainstem Function in Health and Disease* contributed with valuable insight into the multitude of functions involving the brainstem.

## Author Contributions

All authors listed have made a substantial, direct and intellectual contribution to the work, and approved it for publication.

### Conflict of Interest

The authors declare that the research was conducted in the absence of any commercial or financial relationships that could be construed as a potential conflict of interest.

